# Mutational landscape of head and neck squamous cell carcinomas in a South Asian population

**DOI:** 10.1590/1678-4685-GMB-2018-0005

**Published:** 2019-11-14

**Authors:** Kulsoom Ghias, Sadiq S Rehmani, Safina A Razzak, Sarosh Madhani, M. Kamran Azim, Rashida Ahmed, Mumtaz J Khan

**Affiliations:** 1 Department of Biological and Biomedical Sciences, Aga Khan University, Karachi, Pakistan.; 2 Department of Thoracic Surgery, Mount Sinai St. Luke’s Hospital, Icahn School of Medicine at Mount Sinai, New York, NY, USA.; 3 Department of Pathology and Laboratory Medicine, Aga Khan University, Karachi, Pakistan.; 4 Medical College, Aga Khan University, Karachi, Pakistan.; 5 Department of Biosciences, Mohammad Ali Jinnah University, Karachi, Pakistan.; 6 Surgical Specialty Institute, Cleveland Clinic, Abu Dhabi, United Arab Emirates.

**Keywords:** Head and neck squamous cell carcinoma (HNSCC), whole exome sequencing, driver mutation, novel mutation, Pakistani population

## Abstract

Head and neck squamous cell carcinoma (HNSCC) is the sixth most common cancer type globally and contributes significantly to burden of disease in South Asia. In Pakistan, HNSCC is among the most commonly diagnosed cancer in males and females. The increasing regional burden of HNSCC along with a unique set of risk factors merited a deeper investigation of the disease at the genomic level. Whole exome sequencing of HNSCC samples and matched normal genomic DNA analysis (n=7) was performed. Significant somatic single nucleotide variants (SNVs) were identified and pathway analysis performed to determine frequently affected signaling pathways. We identified significant, novel recurrent mutations in *ASNS* (asparagine synthetase) that may affect substrate binding, and variants in driver genes including *TP53, PIK3CA, FGFR2, ARID2, MLL3, MYC* and *ALK*. Using the IntOGen platform, we identified MAP kinase, cell cycle, actin cytoskeleton regulation, PI3K-Akt signaling and other pathways in cancer as affected in the samples. This data is the first of its kind from the Pakistani population. The results of this study can guide a better mechanistic understanding of HNSCC in the population, ultimately contributing new, rational therapeutic targets for the treatment of the disease.

## Introduction

Head and neck squamous cell carcinomas (HNSCC), which include tumours of the oral cavity, oropharynx, hypopharynx and larynx, are the sixth most common cancer worldwide with a global incidence of ~600,000 cases (Jemal *et al.*, 2005, 2011; Hayat *et al.*, 2007; Murar and Forastiere, 2008; Ferlay *et al.*, 2011). In Pakistan, a developing country in South Asia, HNSCC is among the most commonly diagnosed cancers in both males and females (Bhurgri *et al.*, 2006; Masood *et al.*, 2015). The major risk factors for HNSCC include tobacco use, alcohol consumption, and human papilloma virus (HPV) infection (Leemans *et al.*, 2018).

HPV-negative disease accounts for ~80% of the HNSCC cases (Leemans *et al.*, 2011). Unlike developed countries, the incidence of HPV-negative disease has steadily increased in developing countries (Leemans *et al.*, 2011). The increased incidence in both males and females in Pakistan can be attributed to the prevalence of traditional risk factor such as smoking. The use of smokeless tobacco, betel nut, *gutka* (a preparation of crushed areca nut, tobacco, slaked lime and other flavorings) and betel quid or *paan* (a preparation of betel leaf, areca nut and occasionally tobacco) along with its related products are additional risk factors in this part of the world (Gupta and Johnson, 2014; Khan *et al.*, 2014; Li *et al.*, 2014).

HNSCC is associated with considerable disease-related mortality and treatment-related morbidity (Forastiere *et al.*, 2001) and is a major public health concern for Pakistan (Bhurgri *et al.*, 2002; Bhurgri 2004, 2005; Warnakulasuriya 2009; Bray *et al.*, 2012) and worldwide. Despite the advances in all the major treatments for HNSCC including surgery, radiotherapy and chemotherapy, the mortality rate is ~50% (Laramore *et al.*, 1992; Leemans *et al.*, 2018). The existing literature focuses primarily on HNSCC in North American and European populations. There is a dearth of information specific for the South Asian population. The unique set of population-specific risk factors, germline variability and molecular heterogeneity of HNSCC demands a thorough molecular profiling of these tumours in this population in order to understand tumour progression, and identify actionable targets for therapy, leading to improved patient care. The aim of the study described here was to identify the global genetic aberrations underlying HPV-negative HNSCC in the South Asian (Pakistani) population.

## Materials and Methods

### Ethics approval and consent to participate

The Aga Khan University Ethics Review Committee approved the procedures used in collecting and processing of participant material and information (reference #: 1003-Sur/ERC-08). Written informed consent to participate was obtained from all subjects.

### Sample collection

Fresh tumour tissue and matched blood were obtained from treatment-naïve patients undergoing surgical resection of HNSCC primary tumour at the Aga Khan University Hospital in Karachi, Pakistan. Patients with confirmed histological diagnosis of HNSCC were included in this study. At the time of resection, fresh tumour tissue away from the necrotic core measuring at least 0.5 cm^2^ was collected and stored in RNAlater^®^ solution (Thermo Fisher Scientific) at -80 °C till further processing. Formalin-fixed tumour tissue samples were assessed by a histopathologist for tumour content and cellularity based on hematoxylin and eosin (H&E) staining. Seven tumour samples negative for HPV with at least 70% cancer cells and 1 μg (50 ng/μl) of extracted DNA (both tumour as well as genomic DNA) were utilized for whole exome sequencing.

### DNA extraction

Genomic and tumour DNA was extracted in-house using TRIzol® Reagent (Invitrogen, USA) according to manufacturer’s instructions. Tumour DNA was extracted from at least 50 mg of tissue and genomic DNA was extracted from 3-5 mL of peripheral blood samples obtained before patients underwent surgical procedure. DNA yield and quality was assessed both in-house using a NanoDrop 2000 spectrophotometer (Thermo Scientific, USA) and by Macrogen Inc. (Seoul, South Korea) using PicoGreen® Assay (Invitrogen, USA).

### Tumour HPV status

Formalin-fixed paraffin embedded (FFPE) tumour blocks were retrieved and DNA was extracted for assessing HPV status. PCR detection was performed using two sets of general HPV primers (GP5/GP6) (Baay *et al.*, 1996; Khan *et al.*, 2007). Additionally, HPV *in situ* hybridization (ISH) was performed on FFPE blocks using GenPoint assay according to the manufacturer’s instructions (Dako, Denmark). Dako assay can detect HPV-DNA from 13 high-risk genotypes.

### Whole Exome Sequencing (WES)

WES was performed by Macrogen Inc. (Seoul, South Korea). 1-2 μg of tumour and genomic DNA was fragmented by nebulization. DNA libraries were prepared from each sample using TruSeq DNA Sample Prep Kit using the manufacturer’s protocol (Illumina, USA). Unique molecular indices were used for each sample. Exome enrichment was performed using the TruSeq Exome Enrichment kit (Illumina, USA). Paired-end sequencing was performed on Illumina HiSeq 2000 instrument. Each read was of 100 bp size.

### Availability of data and materials

The data sets supporting the results of this article are included within the article and its supplementary files. The raw sequencing data of those patients that consented to deposition of data in a public database (4 out of 7 total) have been deposited in NCBI’s Sequence Read Archive and are accessible through accession number SRP083063.

### Data analysis

Paired-end sequence reads from Illumina were mapped against UCSC Human Genome (hg) 19 using BWA (Li and Durbin 2009). Local realignment was performed using Genome Analysis Tool Kit (GATK) to improve mapping quality (McKenna *et al.*, 2010). Single nucleotide variants (SNVs) were identified in both somatic and germline DNA using MuTect (high-confidence mode) with default settings. Somatic variants were defined as those SNVs which were only identified in the somatic DNA and not seen in germline DNA. Variants marked REJECT were excluded from downstream analysis. Tumour mutational burden was calculated as previously described by others (Chalmers *et al.*, 2017). All mutations were annotated and prioritized using Variant Effect Predictor (VEP) and ANNOVAR. Further characterization of SNVs into missense, nonsense, frameshift, stop loss and stop gain variants was done using wANNOVAR, SNPEFF, SIFT and Polyphen. All somatic missense mutations were analysed for their likely tumourigenic impact based on CHASM (Cancer-specific High-throughput Annotation of Somatic Mutations) (Carter *et al.*, 2009; Wong *et al.*, 2011) and the IntOGen-mutations platform (Gonzalez-Perez *et al.*, 2013). A cut-off score threshold of ≤ 0.2 for FDR with a *p*-value of ≤ 0.05 was applied. The annotation ranked the SNVs for somatic driver mutations for specific cancer tissue types, predicted protein functional impact, allele frequencies from the 1000 Genomes Project and ESP6500 populations, and previous cancer association of the gene harbouring the variants. CHASM training set is composed of a positive class of driver mutations from the COSMIC database and VEST training set comprising a positive class of disease mutations from the Human Gene Mutation Database 66 and a negative class of variants detected in the ESP6500 population and 1000 Genomes Project cohort with an allele frequency of >1%. SNPeff (Cingolani *et al.*, 2012) and CHASM were used to identify stop-gain, start-loss and splice site variants in nonsynonymous coding region. Those SNVs identified by both tools were selected as significant. Mutations in non-coding regions were annotated using CADD and a cut-off threshold score of ≥15 with *p*<10^–5^ applied to predict benign and deleterious variants (Kircher *et al.*, 2014). Pathway analysis was carried out using the IntOGen-Mutations platform (Gonzalez-Perez *et al.*, 2013) and significantly (*p*≤0.05) affected pathways in the cohort and genes within identified.

### ASNS protein modeling

The homology model of human asparagine synthetase was constructed using crystal structural coordinates of the enzyme from Escherichia coli (PDB id 1CT9). The Modeller program (Fiser and Sali, 2003) was used to build the asparagine synthetase model.

## Results

### Clinical characteristics and HPV-status of HNSCC patients

**Figure 1:**
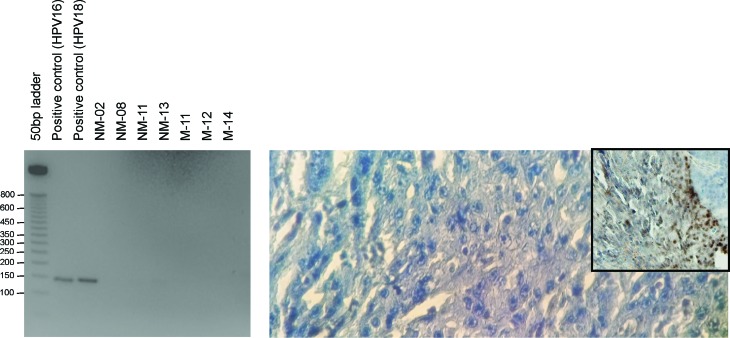
Human papilloma virus (HPV) detection. PCR (left) for HPV detection using GP5/GP6 primers (expected product ~150bp). HPV *in situ* hybridization (right) using GenPoint in a representative HPV-negative HNSCC sample at a magnification of 40 x 10X; inset at magnification of 4 x 10X shows control HPV-positive nuclei stained brown.

Primary tumour samples from 7 treatment-naïve HNSCC patients (Figure S1), along with their matched genomic DNA, were used for this study. The detailed demographics and clinical characteristics of these patients are provided in Table 1. The samples were taken from five male and two female patients, who had an average age at diagnosis of 54 years (SD = 13.24). Two patients reported a family history of cancer; one patient had a personal history of smoking (110 pack years), two of oral tobacco use, one of alcohol and oral tobacco use, and four reported use of betel nut/quid. All samples were negative for human papilloma virus (Figure 1).

**Table 1:** Clinical characteristics of HNSCC patients. Data that is unavailable is indicated with a dash (-).

Sample ID	Gender	Age at diagnosis	Family history of cancer (type)	Smoking history	Oral tobacco use	Betel nut/quid use	Alcohol use	TNM	Stage	Tumour site
NM-02	M	67	Yes (brain)	Yes	No	-	No	pT4N0M0	IV	Left buccal mucosa
				(110 pack years)						
NM-08	M	35	No	No	Yes	Yes	No	pT3N0M0	III	Right buccal mucosa
NM-11	M	57	No	No	No	No	No	pT1N0M0	I	Right tongue
NM-13	F	40	No	No	Yes	Yes	No	pT1N1M0	III	Left tongue
M-11	M	71	Yes (-)	No	Yes	Yes	Yes	pT4N2bM0	IV	Right pyriform fossa
M-12	F	49	No	No	No	Yes	No	T2N2bM0	IV	Lower mandible alveolus
M-14	M	56	No	No	No	No	No	pT3N1M0	III	Right tongue

### Summary of exome capture and sequencing results

**Figure 2:**
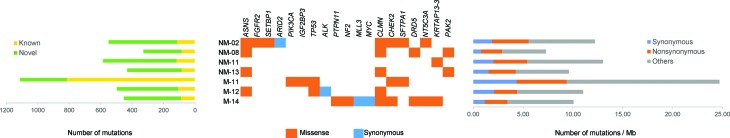
Mutational landscape of HNSCC tumours. Left panel: Number of mutations (known and novel) in HNSCC patients Middle panel: significant somatic nucleotide variants (synonymous, nonsynonymous missense) Right panel: Rate of synonymous, nonsynonymous and other (3’ UTR, 3’ flank, 5’ UTR, 5’ flank, intron, splice site) mutations expressed in mutations per megabase of covered target sequence.

**Figure 3:**
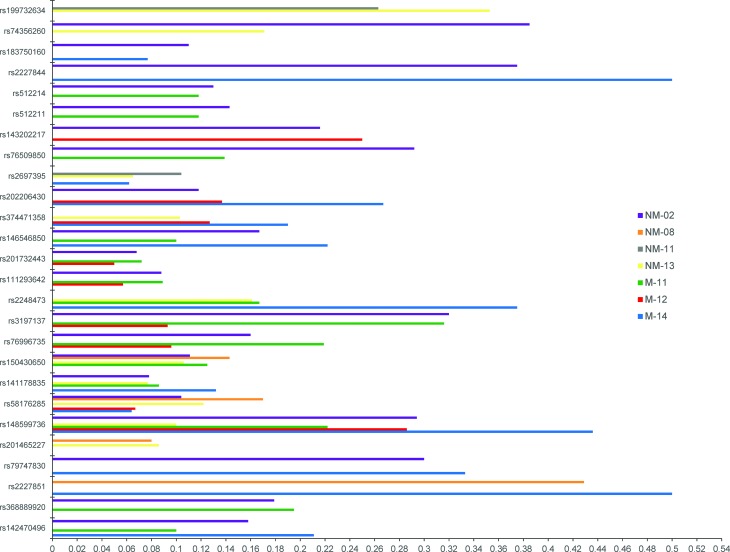
Somatic coding single nucleotide variants (SNV) found in ≥ 2 HNSCC patients and dbSNP database. The variant allele frequency (VAF) on the x-axis indicates the proportion of reads with the variant allele within individual samples.

Paired-end whole exome sequencing (WES) of all seven HNSCC samples and matched genomic DNA was performed on Illumina HiSeq 2000 platform. Each read was of 100 bp size. Additional details of the sequencing, including coverage and depth, are summarized in Table S1. Whole exome sequencing revealed a total of 3,959 single nucleotide variants across all 7 HNSCC samples, of which 2,547 are novel (Figure 2; Table 2, left panel). Nonsynonymous mutation rates ranged from 2.11 to 5.02 mutations per megabase (mean = 3.07) (Table 2, right panel). Several mutations recurred in more than one sample in both coding (Figure 3; Table 3) and non-coding regions (Table S2). Nonsense and splice site variants were also identified in all samples (Table S3).

**Table 2:** Number of somatic single nucleotide variants (SNVs) in HNSCC patients; total and (novel).

Sample ID	NM-02	NM-08	NM-11	NM-13	M-11	M-12	M-14
Nonsynonymous							
Missense	151 (106)	91 (55)	145 (111)	122 (90)	221 (61)	101 (67)	95 (65)
Nonsense	14 (11)	4 (4)	7 (6)	1 (1)	5 (4)	4 (4)	7 (5)
Synonymous	85 (49)	35 (14)	91 (61)	69 (35)	196 (30)	93 (45)	52 (20)
3’ UTR	199 (179)	131 (117)	176 (155)	156 (145)	477 (138)	206 (193)	200 (185)
3’ Flank	34 (32)	23 (20)	52 (49)	24 (23)	78 (25)	35 (31)	40 (37)
5’ UTR	22 (21)	14 (10)	15 (10)	14 (10)	32 (11)	17 (15)	17 (15)
5’ Flank	8 (4)	6 (5)	24 (18)	9 (8)	23 (8)	9 (6)	5 (5)
Intron	33 (32)	23 (17)	74 (56)	35 (33)	79 (20)	29 (26)	34 (30)
Splice site	4 (3)	2 (1)	2 (2)	2 (2)	1 (1)	3 (3)	3 (2)
**Total (novel)**	**550 (437)**	**329 (243)**	**586 (468)**	**432 (347)**	**1112 (298)**	**497 (390)**	**453 (364)**

**Table 3:** Somatic coding single nucleotide variants (SNVs) found in ≥2 HNSCC patients. NS: nonsynonymous; S: synonymous. The variant allele frequency (VAF) indicates the proportion of reads with the variant allele within individual samples.

Gene	Chr	Position	Base change	Amino acid change	Variant type	Variant Allele Frequency (VAF)							Freq	COSMIC ID	rsID
						NM02	NM08	NM11	NM13	M11	M12	M14			
*CLMN*	chr14	95669509	A>G	L726P	NS	0.128	0.118	-	0.145	-	0.079	0.111	5/7	COSM1293528	-
*CHEK2*	chr22	29091840	T>C	K152E	NS	0.158	-	-	-	0.1	-	0.211	3/7	COSM42871	rs142470496
*SFTPA1*	chr10	81373600	G>A	G160S	NS	0.179	-	-	-	0.195	-	-	2/7	-	rs368889920
*DRD5*	chr4	9784542	A>C	T297P	NS	-	0.429	-	-	-	-	0.5	2/7	COSM1431796	rs2227851
*NT5C3A*	chr7	33054388	T>C	D283G	NS	0.3	-	-	-	-	-	0.333	2/7	COSM222478	rs79747830
*KRTAP13-3*	chr21	31797833	C>T	R133K	NS	-	-	0.167	-	-	-	0.089	2/7	-	-
*PAK2*	chr3	196509577	C>G	Q101H	NS	-	0.8	-	0.086	-	-	-	2/7	COSM1422035	rs201465227
*ST6GALNAC4*	chr9	130674582	G>A	-	S	0.294	-	-	0.1	0.222	0.286	0.436	5/7	-	rs148599736
*PPA1*	chr10	71969413	A>G	-	S	0.118	0.143	-	0.109	0.108	-	0.16	5/7	-	-
*KRT83*	chr12	52709871	G>A	-	S	0.173	-	-	0.136	0.147	0.176	0.375	5/7	-	-
*MYLK*	chr3	123419183	G>A	-	S	0.104	0.17	-	0.122	-	0.067	0.064	5/7	-	rs58176285
*OR8I2*	chr11	55861593	G>A	-	S	0.393	0.221	-	0.239	-	0.218	0.261	5/7	-	-
*HIST1H2BL*	chr6	27775319	G>A	-	S	0.078	-	-	0.077	0.086	-	0.132	4/7	-	rs141178835
*ST6GALNAC4*	chr9	130674558	C>T	-	S	-	0.3	-		0.182	0.279	0.488	4/7	-	-
*PPA1*	chr10	71969401	T>C	-	S	0.111	0.143	-	0.106	0.125	-	-	4/7	-	rs150430650
*KRT83*	chr12	52709724	A>G	-	S	0.101	0.097	-	-	0.092	0.093	-	4/7	-	-
*KRT83*	chr12	52709895	G>A	-	S	0.24	-	-	-	0.192	0.245	0.429	4/7	-	-
*OR1M1*	chr19	9204157	T>C	-	S	0.129	-	-	0.071	0.176	0.231	-	4/7	-	-
*OR1M1*	chr19	9204184	G>A	-	S	0.325	-	-	0.113	0.089	0.296	-	4/7	-	-
*HHIPL2*	chr1	222715425	A>G	-	S	0.244	-	-	0.17	-	0.17	0.111	4/7	-	-
*MYLK*	chr3	123419189	C>T	-	S	0.132	0.163	-	0.111	-	-	0.065	4/7	-	-
*ASNS*	chr7	97498451	C>G	-	S	0.16	-	-	-	0.219	0.096	-	3/7	-	rs76996735
*IFITM1*	chr11	315009	C>T	-	S	0.32	-	-	-	0.316	0.093	-	3/7	-	rs3197137
*KRT83*	chr12	52709883	T>C	-	S	-	-	-	0.161	0.167	-	0.375	3/7	-	rs2248473
*OR7D4*	chr19	9324989	C>T	-	S	0.088	-	-	-	0.089	0.057	-	3/7	-	rs111293642
*OR7D4*	chr19	9324995	C>T	-	S	0.068	-	-	-	0.072	0.05	-	3/7	-	rs201732443
*CHEK2*	chr22	29091841	G>A	-	S	0.167	-	-	-	0.1	-	0.222	3/7	-	rs146546850
*OR10G7*	chr11	123908827	T>C	-	S	0.17	-	-	0.074	-	0.069	-	3/7	-	-
*KRT86*	chr12	52699041	G>A	-	S	-	-	-	0.141	-	0.127	0.114	3/7	-	-
*KRT86*	chr12	52699545	G>A	-	S	-	-	-	0.103	-	0.127	0.19	3/7	-	rs374471358
*KRT83*	chr12	52710279	T>C	-	S	0.118	-	-	-	-	0.137	0.267	3/7	-	rs202206430
*KRTAP4-8*	chr17	39254154	C>T	-	S	0.167	-	-	0.173	-	0.102	-	3/7	-	-
*DHX40*	chr17	57663568	A>G	-	S	-	-	0.104	0.065	-	-	0.062	3/7	-	rs2697395
*LCE1E*	chr1	152759892	A>T	-	S	-	0.25	-	-	0.263	-	-	2/7	-	-
*FOLH1*	chr11	49204790	A>G	-	S	0.292	-	-	-	0.139	-	-	2/7	-	rs76509850
*KRT81*	chr12	52681089	G>A	-	S	-	-	-	0.167	0.214	-	-	2/7	-	-
*KRT81*	chr12	52681092	C>T	-	S	-	-	-	0.167	0.214	-	-	2/7	-	-
*KRT83*	chr12	52710790	T>C	-	S	0.216	-	-	-	-	0.25	-	2/7	-	rs143202217
*KRT83*	chr12	52714757	T>C	-	S	-	-	-	0.082	-	0.227	-	2/7	-	-
*KRT83*	chr12	52710798	T>C	-	S	0.235	-	-	-	-	0.268	-	2/7	-	-
*KRT83*	chr12	52713122	C>T	-	S	-	-	-	0.179	-	0.113	-	2/7	-	-
*SEH1L*	chr18	12955467	T>C	-	S	-	-	-	-	0.086	0.128	-	2/7	-	-
*KRTAP10-7*	chr21	46020536	T>C	-	S	0.143	-	-	-	0.118	-	-	2/7	-	rs512211
*KRTAP10-7*	chr21	46020542	T>C	-	S	0.13	-	-	-	0.118	-	-	2/7	-	rs512214
*HIST2H2AC*	chr1	149858563	C>T	-	S	-	-	-	-	-	0.096	0.093	2/7	-	-
*HIST2H2AC*	chr1	149858593	C>T	-	S	-	-	-	-	-	0.125	0.071	2/7	-	-
*KIAA1549*	chr7	138601891	A>T	-	S	0.063	-	-	-	-	0.098	-	2/7	-	-
*GIMAP5*	chr7	150439323	A>G	-	S	-	-	-	-	-	0.115	0.278	2/7	-	-
*KRTAP5-11*	chr11	71293458	G>A	-	S	-	-	-	0.093	-	0.095	-	2/7	-	-
*OR10G8*	chr11	123901199	G>A	-	S	-	-	-	0.118	-	0.074	-	2/7	-	-
*OR10G8*	chr11	123901211	G>A	-	S	-	-	-	0.136	-	0.12	-	2/7	-	-
*DUOX1*	chr15	45433188	T>C	-	S	0.071	-	-	-	-	0.081	-	2/7	-	-
*KRTAP4-11*	chr17	39274373	G>A	-	S	-	-	-	-	-	0.049	0.111	2/7	-	-
*KRTAP4-12*	chr17	39280045	G>A	-	S	-	-	-	0.14	-	0.145	-	2/7	-	-
*KRTAP10-4*	chr21	45994676	A>G	-	S	0.103	-	-	-	-	0.065	-	2/7	-	-
*HIST2H2AB*	chr1	149859383	C>T	-	S	-	-	-	0.118	-	-	0.071	2/7	-	-
*DRD5*	chr4	9784550	G>A	-	S	0.375	-	-	-	-	-	0.5	2/7	-	rs2227844
*KRTAP5-5*	chr11	1651760	C>T	-	S	0.11	-	-	-	-	-	0.077	2/7	-	rs183750160
*DPY19L2*	chr12	63964600	G>A	-	S	-	-	-	0.103	-	-	0.105	2/7	-	-
*SETD8*	chr12	123875311	C>T	-	S	0.385	-	-	0.171	-	-	-	2/7	-	rs74356260
*POTEE*	chr2	132021452	C>A	-	S	-	0.176	-	0.094	-	-	-	2/7	-	-
*CBWD1*	chr9	163985	A>G	-	S	-	0.065	0.062	-	-	-	-	2/7	-	-
*ZNF814*	chr19	58385762	C>G	-	S	-	-	0.263	0.353	-	-	-	2/7	-	rs199732634

### Mutational landscape in HNSCC patients

On average, 227 coding mutations were identified per tumour, 39% of which are synonymous. The majority of the mutations identified were nonsynonymous missense mutations and mutations in the 3’ UTR region (Table 2). Filtering for driver and other significant variants using CHASM revealed alterations in genes that have been implicated in HNSCC or other cancers (Figure 2, middle panel; Table 4). Driver missense mutations in *FGFR2* (Fibroblast Growth Factor Receptor 2)*, SETBP1* (SET Binding Protein 1)*, PIK3CA* (Phosphatidylinositol-4,5-Bisphosphate 3-Kinase Catalytic Subunit Alpha)*, IGF2BP3* (Insulin Like Growth Factor 2 MRNA Binding Protein 3)*, TP53* (Tumour Protein P53)*, PTPN11* (Protein Tyrosine Phosphatase, Non-Receptor Type 11) and *NF2* (Neurofibromin 2) were identified. Significant missense mutations were also identified in *ASNS* (Asparagine Synthetase (Glutamine-Hydrolyzing)) in four of the seven samples. Other genes that exhibited recurrent mutations included the *CLMN* (Calmin) gene (5/7), *CHEK2* (Checkpoint Kinase 2) (3/7), and *DRD5* (Dopamine Receptor D5) and *PAK2* (P21 (RAC1) Activated Kinase 2) (2/7) (Table 3). These recurrent mutation sites have not been reported as hotspots in previous HNSCC sequencing studies.

**Table 4:** Somatic single nucleotide variants (SNVs) in HNSCC patients in coding regions. NS MS: nonsynonymous missense; S: synonymous. Driver missense variants are in bold text and synonymous variants in possible driver genes are marked with an asterisk (*).The variant allele frequency (VAF) indicates the proportion of reads with the variant allele within individual samples. The minor allele frequency (MAF) signifies prevalence of the known variants in the global population as per the ExAc dataset.

Sample ID	Gene	Chr	Position	Variant type	Base change	Amino acid change	Variant allele frequency	Minor allele frequency	rsID	COSMIC ID
NM-02	*FGFR2*	chr10	123256128	NS MS	G>T	P595H	0.273	-	-	-
	*SETBP1*	chr18	42530740	NS MS	G>T	G479C	0.13	-	-	-
	*ASNS*	chr7	97498395	NS MS	G>A	A25V	0.225	-	-	-
		chr7	97498404	NS MS	A>G	M22T	0.243	-	-	-
	*KIF21B*	chr1	200954042	NS MS	G-T	R1250S	0.143	-	-	-
	*UBA7*	chr3	49848502	NS MS	G>T	P382H	0.132	-	-	-
	*KDM3B*	chr5	137735569	NS MS	G>T	A1023S	0.158	-	-	-
	*EXOSC1*	chr10	99196233	NS MS	G>T	A186D	0.3	-	-	-
	*DENND5A*	chr11	9166573	NS MS	C>A	V1031F	0.273	-	-	-
	*DGKZ*	chr11	46394214	NS MS	G>T	G541V	0.5	-	-	
	*FOLH1*	chr11	49186320	NS MS	G>C	N459K	0.227	0.00003	rs201724751	-
		chr11	49204779	NS MS	C>T	R281H	0.3	0.0351	rs116795343	-
	*FAT3*	chr11	92087697	NS MS	G>T	G807C	0.211	-	-	-
	*SLC7A7*	chr14	23282391	NS MS	G>T	L73M	1	-	-	-
	*CEP128*	chr14	81244269	NS MS	A>T	L778Q	0.174	-	-	-
	*EML2*	chr19	46130008	NS MS	C>A	W433C	0.3	-	-	-
	*CHRNA4*	chr20	61981122	NS MS	C>A	K547N	0.375	-	-	-
	*GART*	chr21	34889834	NS MS	C>T	R595Q	0.2	0.0003	rs202015633	-
	*CHEK2*	chr22	29091840	NS MS	T>C	K416E	0.158	0.0259	rs74751600	-
	ARID2*	chr12	46245344	S	G>T	S1146S	0.333	-	-	-
NM-08	*ASNS*	chr7	97498378	NS MS	C>T	A31T	0.167	-	-	-
	*ZFHX4*	chr8	77618158	NS MS	G>T	G612V	0.231	-	-	COSM73358
	*CKAP5*	chr11	46819413	NS MS	C>G	C427S	0.12	-	-	-
	*SF1*	chr11	64537028	NS MS	C>A	R303L	0.097	-	-	-
	*HECTD4*	chr12	112669460	NS MS	C>G	K1885N	0.158	-	-	-
	*FBN1*	chr15	48744840	NS MS	C>T	A1822T	0.273	0.00003	rs777539060	-
	*HELZ*	chr17	65144830	NS MS	G>T	L826I	0.2	-	-	-
NM-11	*RALGPS2*	chr1	178855145	NS MS	C>T	T361M	0.125	-	-	-
	*DYNC1I2*	chr2	172584439	NS MS	C>A	P369T	0.125	-	-	-
	*NFE2L2*	chr2	178098966	NS MS	C>A	D27Y	0.188	-	-	-
	*POSTN*	chr13	38154051	NS MS	G>T	P536Q	0.111	-	-	-
	*INO80*	chr15	41377611	NS MS	G>A	R277C	0.158	-	-	-
	*CDH16*	chr16	66949240	NS MS	G>T	P156T	0.364	-	-	-
	*PRPSAP2*	chr17	18785908	NS MS	T>C	L147S	0.098	-	-	-
NM-13	*ASNS*	chr7	97498378	NS MS	C>T	A31T	0.125	-	-	-
	*ASNS*	chr7	97498395	NS MS	G>A	A25V	0.105	-	-	-
	*GIGYF2*	chr2	233684687	NS MS	C>T	R862C	0.138	0.00002	rs561616045	-
	*CBLB*	chr3	105464767	NS MS	G>T	P280H	0.097	-	-	-
	*LAP3*	chr4	17598708	NS MS	C>A	A343D	0.214	-	-	-
	*TRIM7*	chr5	180625732	NS MS	G>A	L316F	0.156	-	-	-
	*ABCB8*	chr7	150733032	NS MS	G>A	A331T	0.227	0.00004	rs777741819	-
	*ESRP1*	chr8	95674755	NS MS	G>C	V206L	0.079	-	-	-
	*ADCY6*	chr12	49176793	NS MS	C>A	R142L	0.375	-	-	-
	*NFATC4*	chr14	24843541	NS MS	C>T	S581L	0.25	-	-	COSM3793625
	*EML5*	chr14	89124732	NS MS	C>A	G1226W	0.143	-	-	-
	*ANKFY1*	chr17	4086708	NS MS	G>T	A688E	0.176	-	-	-
	*UQCRFS1*	chr19	29698630	NS MS	C>A	C217F	0.25	-	-	-
	*ITSN1*	chr21	35237479	NS MS	G>T	M1305I	0.667	-	-	-
	*ABCB7*	chrX	74332770	NS MS	C>G	C96S	0.111	-	-	-
	*HDX*	chrX	83730396	NS MS	G>C	R4G	0.231	-	-	-
M-11	*PIK3CA*	chr3	178936091	NS MS	G>A	E545K	0.278	0.000008	rs104886003	COSM763
	*IGF2BP3*	chr7	23353160	NS MS	A>G	I503T	0.211	0.0040	rs79900450	-
	*TP53*	chr17	7577106	NS MS	G>C	P278A	0.647	-	-	COSM10814
	*SLC8A1*	chr2	40656504	NS MS	C>T	G306D	0.234	-	-	-
	*MITF*	chr3	70008494	NS MS	C>A	Q362K	0.333	-	-	-
	*VEPH1*	chr3	157034861	NS MS	A>G	L622P	0.139	-	-	-
		chr3	157099046	NS MS	C>G	L342F	0.208	-	-	-
	*GNGT1*	chr7	93536114	NS MS	T>C	V19A	0.133	-	-	-
	*ARHGEF10*	chr8	1824752	NS MS	A>G	D232G	0.214	-	-	-
	*NEBL*	chr10	21098782	NS MS	T>A	D855V	0.339	-	-	-
	*NAALAD2*	chr11	89891404	NS MS	A>C	L296F	0.375	-	-	-
	*SMG8*	chr17	57290439	NS MS	A>T	H752L	0.25	-	-	
	*RBM39*	chr20	34302295	NS MS	C>A	C303F	0.15	-	-	-
M-12	*ASNS*	chr7	97498378	NS MS	C>T	A31T	0.214	-	-	-
	*GRK7*	chr3	141499490	NS MS	A>C	Y296S	0.273	-	-	-
	*TIPARP*	chr3	156413805	NS MS	C>A	P413Q	0.121	-	-	-
	*RGS3*	chr9	116346401	NS MS	C>A	S903R	1	-	-	-
	*SPTBN2*	chr11	66472616	NS MS	C>A	G711C	1	-	-	-
	*UBE4A*	chr11	118253450	NS MS	C>A	A726E	0.15	-	-	-
	*TP53*	chr17	7577538	NS MS	C>T	R248Q	0.286	0.00006	rs11540652	COSM10662
	*CDC27*	chr17	45229257	NS MS	T>C	T335A	0.167	0.00002	rs199890121	-
	*HELZ*	chr17	65163619	NS MS	C>A	C575F	0.667	-	-	-
	ALK*	chr2	29474099	S	C>A	G692G	1	-	-	-
M-14	*PTPN11*	chr12	112892407	NS MS	T>G	S189A	0.167	0.0027	rs79068130	-
	*NF2*	chr22	30090766	NS MS	G>T	R588L	1	-	-	-
	MLL3*	chr7	151962176	S	T>A	P377P	0.084	0.4554	rs62478356	COSM4162022
	MYC*	chr8	128750817	S	C>A	T118T	0.5	-	-	-

Synonymous variants in previously identified driver genes *ARID2* (AT-Rich Interaction Domain 2)*, ALK* (Anaplastic Lymphoma Receptor Tyrosine Kinase)*, MLL3* [Myeloid/Lymphoid Or Mixed-Lineage Leukemia 3, also known as *KMT2C* (Lysine Methyltransferase 2C)] and *MYC* (V-Myc Avian Myelocytomatosis Viral Oncogene Homolog), were also identified (Figure 2, middle panel; Table 4). The *ASNS* gene was found to have a synonymous mutation in three samples, and recurrent synonymous mutations were also observed in *CHEK2* and *DRD5* genes (Table 3). Splice site variants in *FCGR2A* (Fc Fragment of IgG, Low Affinity IIa, Receptor (CD32)) and two genes involved in eukaryotic translation initiation [*EIF4B* (Eukaryotic Translation Initiation Factor 4B) and *EIF4A3* (Eukaryotic Translation Initiation Factor 4A3)] were seen in two of the seven samples (Table S3). Significant non-coding mutations were filtered using CADD (Table S4). In the 3’UTR region, mutations in *IGF1R* (Insulin Like Growth Factor 1 Receptor) and *ERBB4* (Erb-B2 Receptor Tyrosine Kinase 4) were identified as significant. Another eukaryotic translation initiation factor, *EIF2B4* (Eukaryotic Translation Initiation Factor 2B Subunit Delta), exhibited significant splice site variance. IntOGen pathway analysis revealed that the MAP kinase pathway was the most significantly affected pathway in all samples tested. In addition, cell cycle, actin cytoskeleton regulation, PI3K-Akt signaling and other pathways in cancer were among those significantly enriched for exomic alterations in all samples (Table 5). Genes with driver mutations implicated in multiple pathways included *FGFR2, PIK3CA,* and *TP53*. Significant mutations in the pathway genes were all deleterious with respect to protein function as predicted by SIFT and PolyPhen.

**Table 5:** Significantly involved pathways (*p* ≤ 0.05) identified by IntOGen-Mutations platform**.** Driver mutations in each pathway are in bold text and marked with an asterisk (*).

Pathway ID	KEGG annotation	Total genes in pathway	Number of genes affected	Pathway genes with significant/ driver (*) mutations
hsa04010	MAPK signaling pathway	257	46	FGFR2*
				PIK3CA*
				TP53*
hsa04110	Cell cycle	124	35	TP53*
				*CHEK2*
				*CDC27*
hsa05166	HTLV-1 infection	260	60	PIK3CA*
				TP53*
				*CHEK2*
				*CDC27*
				*ADCY6*
				*NFATC4*
hsa05200	Pathways in cancer	326	71	FGFR2*
				PIK3CA*
				TP53*
				*CBLB*
				*ADCY6*
				*ADCY6*
				*GNGT1*
				*MITF*
hsa04810	Regulation of actin cytoskeleton	213	46	FGFR2*
				PIK3CA*
hsa04151	PI3K-Akt signaling pathway	338	80	*FGFR2*
				PIK3CA*
				TP53*
				*GNGT1*

### Asparagine synthetase protein modeling

**Figure 4:**
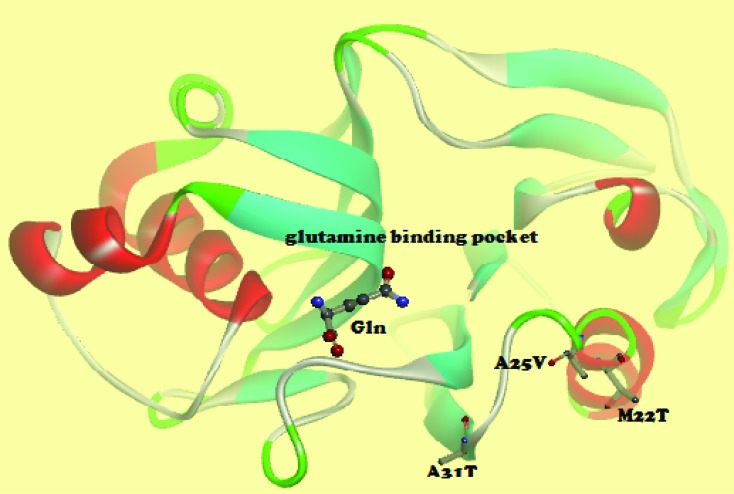
Homology model of the N-terminal domain of human asparagine synthetase (ASNS) complexed with glutamine (Gln). Amino acid changes due to nonsynonymous mutations in *ASNS* are indicated.

The *ASNS* gene codes for asparagine synthetase, which catalyzes the formation of asparagine from glutamine, aspartate and ATP. Protein modeling of the effect of the three novel, recurrent mutations in *ASNS* identified in this cohort revealed that the mutated amino acids (p.A13T, p.A25V and p.M22T) are located in the vicinity (within 10 Å distance) of the glutamine binding pocket (Figure 4).

## Discussion

This is the first study reported in the literature to describe the mutational landscape of Pakistani HNSCC patients. We performed exome sequencing of a small set of HPV-negative HNSCC patients from Pakistan. We identified a total of ~4000 somatic variants (novel and known). Previous studies have reported greater number of mutations in HPV-negative as compared to HPV-positive HNSCC tumours (Riaz *et al.*, 2014; Beck and Golemis, 2016). As a comparison, Stransky *et al.* (2011) on average found 130 coding mutations per tumour (25% synonymous), while in the current cohort an average of 227 coding mutations per tumour (39% synonymous) were identified.

Several variants were found in more than one sample and in genes that have been previously identified to play a role in HNSCC carcinogenesis. Next generation sequencing studies in other populations have identified mutations in the tumour suppressor gene *TP53*, which is associated with smoking-related disease, and the oncogene *PIK3CA,* at a mutation rate of 40-60% and 6-8%, respectively (Agrawal *et al.*, 2011; Stransky *et al.*, 2011; Loyo *et al.*, 2013). The TCGA study, with the largest cohort to date, reported a *TP53* mutation rate of 72% and *PIK3CA* mutation rate of 18-21% (TCGA 2015). Mutations in *TP53* gene were detected in two of the seven cases in the current study, and in *PIK3CA* in one patient. In a comparative genomic analysis of HPV-positive and HPV-negative tumours, the former showed mutations in *FGFR2* and *MLL3*, among others. The mutational spectrum in HPV-negative tumours closely resembled lung and esophageal squamous cell carcinomas, with mutations identified in genes including *TP53, MLL2/3, NOTCH1, PIK3CA* and *DDR2* (Seiwert *et al.*, 2015). The HPV-negative cohort in the current study exhibited a nonsense variant (p.Y223X) in *DDR2* in a single sample. A different nonsense mutation (p.R709X) and missense mutations (p.I474M; p.I724M) have been previously identified exclusively in HNSCC recurrences (Hedberg *et al.*, 2015). *DDR2* and *FGFR2*, which was identified as having a potential missense driver mutation in one sample in the current study, are both genes that code for receptor tyrosine kinases and are potentially targetable for therapeutics. In addition, an SNV was identified in *MLL3* in a sample that also exhibited an SNV in the driver gene *MYC*. *MLL* genes encode histone lysine methyltransferases that are involved in chromatin remodeling. Recurrent mutations in *MLL* genes have been identified in several other cancers, including lung squamous cell carcinoma, and been associated with poor clinical outcomes (Morin *et al.*, 2011; Grasso *et al.*, 2012; Jones *et al.*, 2012; Kim *et al.*, 2014; Seiwert *et al.*, 2015). The oncogene *MYC* is most often altered in HPV-negative HNSCC tumours (TCGA 2015).

Additionally, we discovered recurrent significant missense mutations in *ASNS* (asparagine synthetase) gene in 4 out of 7 samples. These SNVs in *ASNS* have not previously been reported in the literature as significant in HNSCC pathogenesis. The *ASNS* gene codes for a ubiquitously expressed, ATP-dependent enzyme that converts aspartate and glutamine to asparagine and glutamate (Balasubramanian *et al.*, 2013). The protein folds into two distinct domains, where the N-terminal domain contains two layers of antiparallel beta-sheets. The active site responsible for the binding and hydrolysis of glutamine is situated between these layers and important, evolutionarily conserved side chains involved in glutamine binding within the substrate binding pocket include Arg 49, Asn 74, Glu 76, and Asp 98 (Van Heeke and Schuster, 1989). While the amino acids mutated as a result of the novel and recurrent mutations in *ASNS* identified in this cohort are not part of the glutamine binding pocket, protein modeling revealed their proximity to the region. Therefore, these mutations may affect glutamine binding during catalysis. Elevated levels of ASNS play a role in drug resistance in acute lymphoblastic leukemias and have been implicated in solid tumour adaptation to nutrient deprivation and hypoxia (Balasubramanian *et al.*, 2013). ASNS expression has also been shown to be an independent factor affecting survival in hepatocellular carcinoma and low ASNS levels are correlated with poorer surgical outcomes (Zhang *et al.*, 2013). In HNSCC, deregulation of miR-183-5p and its target gene *ASNS* has been documented in a radiochemotherapy cell culture model of primary HNSCC cells and is a potential prognostic marker for radiochemotherapy outcome (Summerer *et al.*, 2015). Two recent reports have further elucidated the role of ASNS in carcinogenesis. On*e* showed that ASNS expression in primary tumours is correlated with metastatic relapse and bioavailability of asparagine regulates metastatic potential and progression in breast cancer cells, potentially by affecting the epithelial-to-mesenchymal transition (Knott *et al.*, 2018). ASNS was also identified as a key target of the KRAS-ATF4 axis in non-small-cell lung cancer. Oncogenic KRAS regulates amino acid homeostasis and cellular response to nutrient stress via the ATF4 target ASNS, which subsequently contributes to inhibition of apoptosis and increase in proliferation of cancer cells (Gwinn *et al.*, 2018). While *KRAS* mutations are uncommon in HNSCC, particularly as compared to *HRAS* (Rothenberg and Ellisen, 2012), mutations in *ASNS* could effectively have the same functional consequences. Given the role of *ASNS* in cellular stress and the unfolded protein response, it is an intriguing target for further study in HNSCC pathogenesis.

The current analysis also revealed significant low-frequency driver mutations in *SETBP1*, *IGF2BP3*, *PTPN11* and *NF2*. *SETBP1* was identified in a patient who also had a driver mutation in *FGFR2*. *SETBP1* encodes a nuclear protein and its overexpression results in inhibition of the tumour-suppressor PP2A serine-threonine phosphatase activity (Cristobal *et al.*, 2010). Mutations in SETBP1 resulting in overexpression or gain of function have been documented previously in hematological malignancies (Ciccone *et al.*, 2015).

An *IGF2BP3* mutation was found in a sample that also had driver mutations in *PIK3CA* and *TP53*. The protein product of *IGF2BP3* is an RNA-binding factor that promotes cancer invasion by binding to transcripts that encode proteins, such as CD44, for functions related to cell migration, proliferation and adhesion (Ennajdaoui *et al.*, 2016). *IGF2BP3* mutations and copy number variations have been reported previously in HNSCC (Lin *et al.*, 2011; Clauditz *et al.*, 2013; Jimenez *et al.*, 2015), and its role in cell invasiveness and metastasis in several other cancers has been documented in the literature (Schaeffer *et al.*, 2010; Lin *et al.*, 2011; Taniuchi *et al.*, 2014; Hsu *et al.*, 2015; Shantha Kumara *et al.*, 2015; Belharazem *et al.*, 2016; de Lint *et al.*, 2016; Ennajdaoui *et al.*, 2016).

Mutations in *PTPN11* and *NF2* genes were found in the same sample. The protein encoded by the proto-oncogene *PTPN11* is a cytoplasmic tyrosine phosphatase, which is widely expressed in most tissues and known to play a regulatory role in normal hematopoiesis, and in mitogenic activation, metabolic control, transcription regulation, and cell migration signaling pathways (Chan and Feng, 2007). Somatic *PTPN11* mutations have been detected in juvenile myelomonocytic leukemia, myelodysplastic syndromes and acute myeloid leukemia (Tartaglia *et al.*, 2003; Chan and Feng, 2007). While *PTPN11* mutations have not been reported previously in HNSCC, this gene has been identified as a target of the tumour-suppressive microRNA miR-489. Knockdown of *PTPN11* in HNSCC cell lines resulted in the inhibition of cell proliferation (Kikkawa *et al.*, 2010). Neurofibromatosis type 2 (NF2) is a tumour suppressor gene on chromosome 22q12 that encodes for merlin, a membrane-cytoskeleton scaffolding protein that inhibits key signaling pathways crucial to cell proliferation, such as the PI3K pathway. Somatic NF2 mutations have been reported in a number of different cancers (Schroeder *et al.*, 2014). In HNSCC, chromosome 22q is a frequent site of allele loss. Merlin and the cytoplasmic tail of CD44, which is regulated at the transcript level by *IGF2BP3* gene product as mentioned above, create a molecular switch complex that is responsible for either cell growth or proliferation (Morrison *et al.*, 2001).

In addition to non-synonymous mutations, synonymous mutations are known to frequently act as driver mutations in cancers (Supek *et al.*, 2014). We identified SNVs in *MLL3*, *ARID2* and *ALK*. Mutations in MLL and ARID gene families have been previously documented for HNSCC (India Project Team of the International Cancer Genome Consortium, 2013; Martin *et al.*, 2014). The *ALK* gene encodes yet another receptor tyrosine kinase, which has been found to be aberrantly expressed in several tumours, including anaplastic large cell lymphomas (Chiarle *et al.*, 2008; Salaverria *et al.*, 2008), neuroblastoma (Lasorsa *et al.*, 2016; Theruvath *et al.*, 2016; Ueda *et al.*, 2016) and non-small cell lung cancer (Soda *et al.*, 2007; Quere *et al.*, 2016).

A study of gingivo-buccal oral squamous cell carcinoma (OSCC-GB), an HNSCC clinical sub-type, in the Indian population revealed frequently altered genes that are specific to OSCC-GB and others that are also affected in HNSCC (India Project Team of the International Cancer Genome Consortium, 2013). Altered genes that are common between the OSCC-GB study and the current study in the Pakistani population are *ARID2* and *TP53*. MLL family member *MLL4* was also identified as a frequently altered gene specific to OSCC-GB. Other genes identified in the study in the Indian population, such as *CASP8, HRAS* and *NOTCH1*, are also altered in HNSCC in other populations (albeit at different frequencies and with varying significance) (Agrawal *et al.*, 2011; Stransky *et al.*, 2011; Seiwert *et al.*, 2015; The Cancer Genome Atylas Network, 2015; Al-Hebshi *et al.*, 2016), but were not identified in this study.

The small sample size is a limitation of this study, which may explain low frequency of commonly mutated genes and why some of the commonly occurring HNSCC mutations such as *NOTCH1* and *HRAS* were not identified in this small cohort. However, given limited resources, it was deemed important to establish preliminary data prior to a larger scale study. The approach of using a smaller discovery cohort followed by validation of identified mutations in a larger cohort has been proposed and taken by others and reported in the literature (Bacchetti *et al.*, 2011; Nichols *et al.*, 2012; Romero Arenas *et al.*, 2014; Hedberg *et al.*, 2015). It is also possible that given the heterogeneous nature of this disease and unique set of risk factors compared to Western countries, the predominant driver gene mutations may vary among populations. Previous studies in East and South Asian populations with oral squamous cell carcinoma have highlighted that the pattern of genetic mutations is significantly different from tumour profiles in other studies largely conducted in Caucasian populations (Vettore *et al.*, 2015; Su *et al.*, 2017). Population-based differences in mutational profile have also been documented for other cancer types. In lung cancers, several studies have highlighted the geographic variations in genes such as EGFR and LKBI between Asian (Chinese, Japanese, Korean) and Caucasian populations (Koivunen *et al.*, 2008; Mitsudomi, 2014; Li *et al.*, 2015).

This is the first report describing the mutational spectrum of Pakistani HNSCC patients. In addition to reporting known HNSCC mutations, we have identified novel, recurrent mutations in *ASNS* and other genes in the Pakistani population. It has been well established that a complex interplay of genetic and environmental factors results in varying risk of cancer development and treatment outcomes across different ethnicities and geographic regions (Ma *et al.*, 2010). Such diversity among different populations can be explained by the type and frequency of variations in both germline and somatic genomes (Wang and Wheeler, 2014). Therefore, this study is an important step towards gaining a better mechanistic understanding of the complex nature of HNSCC. Future studies will be undertaken to confirm and validate the findings from this study in a larger cohort. Additionally, functional analysis of mutations and correlation with clinical outcomes will be performed.

## Supplementary material

The following online material is available for this article

Figure S1 HNSCC samples.

Table S1 Whole exome sequencing data summary.

Table S2 Somatic non-coding single nucleotide variants (SNVs) found in ≥ 2 HNSCC patients.

Table S3 Genes with somatic mutations in HNSCC patients resulting in nonsense or splice site variants.

Table S4 Significant single nucleotide variant (SNV) mutations in HNSCC patients in non-coding regions.

*Associate Editor: Ricardo G. Correa*
