# Supplementary material for: Mutational landscape of head and neck squamous cell carcinomas in a South Asian population
Source: Genet Mol Biol. 2019 Nov 14;42(3):526–42. doi: 10.1590/1678-4685-GMB-2018-0005 (PMC6905448; doi:10.1590/1678-4685-GMB-2018-0005)
Supplement: Supplementary file 1 [file 1415-4757-GMB-42-3-2018-0005-suppl1.pdf]

Supplementary Material to "Mutational landscape of head and neck squamous cell carcinomas in a South Asian population"

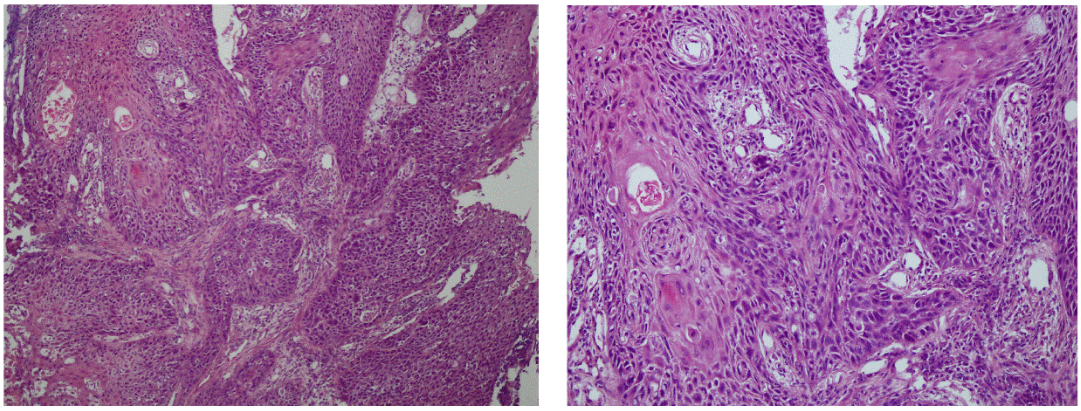

Figure S1 - HNSCC samples.
Hematoxylin and Eosin (H&E) stained representative HBSCC sample with >75 tumor content at magnification of 10x 10X (left) and 40 x 10X (right).
