# Supplementary material for: Mutational landscape of head and neck squamous cell carcinomas in a South Asian population
Source: Genet Mol Biol. 2019 Nov 14;42(3):526–42. doi: 10.1590/1678-4685-GMB-2018-0005 (PMC6905448; doi:10.1590/1678-4685-GMB-2018-0005)
Supplement: Supplementary file 2 [file 1415-4757-GMB-42-3-2018-0005-suppl2.pdf]

Supplementary Material to "Mutational landscape of head and neck squamous cell carcinomas in a South Asian population"

Table S1 - Whole exome sequencing data summary.

	Sample ID	Tumour/ Normal	Total Reads	Mappable Reads	%Mappable Reads	On-target Reads	%on-target reads (out of mappable)	%on-target reads (out of total)	%Cov (>1X)	%Cov (>10X)	Med. Depth	Mean Depth
	NM-02	T	64479832	37991627	58,92%	21158316	55,69%	32,81%	93,50%	67,90%	18	23,9
	NM-08	T	66605108	38757591	58,19%	21459727	55,37%	32,22%	92,60%	65,30%	17	24
	NM-11	T	65275076	31909069	48,88%	17049648	53,43%	26,12%	86,90%	54,80%	13	19,5
	NM-13	T	66844732	40290161	60,27%	23128810	57,41%	34,60%	94,30%	74,60%	21	26,6
	M-11	T	60485306	33127379	54,77%	18397514	55,54%	30,42%	92,70%	61,40%	15	20,9
	M-12	T	58426468	34550935	59,14%	19809662	57,33%	33,91%	93,70%	69,00%	17	22,6
	M-14	T	57213860	34677127	60,61%	20002119	57,68%	34,96%	93,60%	66,90%	17	22,9
**Mean**			**62761483**	**35900556**	**57,25%**	**20143685**	**56,06%**	**32,15%**	**92,50%**	**65,70%**	**--**	**22,9**
**Median**			**64479832**	**34677127**	**58,92%**	**20002119**	**55,69%**	**32,81%**	**93,50%**	**66,90%**	**17**	**22,9**
	NM-02	N	59444834	43665786	73,46%	28837727	66,04%	48,51%	94,10%	83,90%	33	37,6
	NM-08	N	64999028	57420941	88,34%	38582326	67,19%	59,36%	95,50%	88,00%	46	50,7
	NM-11	N	107609428	71586350	66,52%	49638587	69,34%	46,13%	94,50%	87,10%	58	64,6
	NM-13	N	67960688	49317492	72,57%	33388295	67,70%	49,13%	94,20%	86,30%	38	43,1
	M-11	N	62461258	44276283	70,89%	31794725	71,81%	50,90%	94,20%	86,60%	38	40,4
	M-12	N	157859610	117125279	74,20%	78998627	67,45%	50,04%	95,90%	91,90%	99	103,8

	Sample ID	Tumour/ Normal	Total Reads	Mappable Reads	%Mappable Reads	On-target Reads	%on-target reads (out of mappable)	%on-target reads (out of total)	%Cov (>1X)	%Cov (>10X)	Med. Depth	Mean Depth
	M-14	N	64299060	46073206	71,65%	30112797	65,36%	46,83%	94,50%	85,70%	35	39
**Mean**			**83519129**	**61352191**	**73,95%**	**41621869**	**67,84%**	**50,13%**	**94,70%**	**87,10%**	**--**	**54,17**
**Median**			**64999028**	**49317492**	**72,57%**	**33388295**	**67,45%**	**49,13%**	**94,50%**	**86,60%**	**38**	**43,1**
