# Supplementary material for: Mutational landscape of head and neck squamous cell carcinomas in a South Asian population
Source: Genet Mol Biol. 2019 Nov 14;42(3):526–42. doi: 10.1590/1678-4685-GMB-2018-0005 (PMC6905448; doi:10.1590/1678-4685-GMB-2018-0005)
Supplement: Supplementary file 3 [file 1415-4757-GMB-42-3-2018-0005-suppl3.pdf]

Supplementary Material to "Mutational landscape of head and neck squamous cell carcinomas in a South Asian population"

Table S2 - Somatic non-coding single nucleotide variants (SNVs) found in ≥2 HNSCC patients.

Sample ID	Gene	Base change	rsID	Frequency (out of 7 total samples)
3' UTR	*ZBTB44*	T>G		7
	ELSPBP1	C>T		
	LEP	A>G		6/7
	KLRD1	G>A		
	ELSPBP1	G>T		
	SPATA5	T>C		5/7
	ORAI2	T>C		
	KLRD1	T>C		
	ARF4	G>A	rs201857922	4/7
	LEP	C>T	rs199839608	
	KLRD1	G>T		
	ATXN3	T>A		
	HJURP	A>G		
	SSR3	A>C		
	SPATA5	T>A		
	HIST1H4J	T>C	rs2277104	
	HIST1H4J	C>T		

**Sample ID**	**Gene**	**Base change**	**rsID**	**Frequency (out of 7 total samples)**
	SMU1	T>A		
	SFXN2	C>T		
	FLCN	C>T		
	SSR3	G>A		
	AGMAT	A>G		
	ZBTB8A	G>C		
	TMEM128	T>G	rs78934832	
	NDST3	A>G		
	SVOP	T>G		
	SNX6	C>A		
	SNX6	C>G		
	DNAL1	T>C		
	USP14	A>G		
	AXL	A>G		3/7
	FAM134A	G>T		
	NDST3	A>G		
	CARD11	G>A	rs1636175	
	PSPH	C>T	rs144707333	
	PSPH	A>C		
	CPA4	G>T		
	SFXN2	G>A		
	MDM2	G>C		
	TEP1	T>C		

Sample ID	Gene	Base change	rsID	Frequency (out of 7 total samples)
	TEP1	A>C		
	CLTC	T>C		
	NFAM1	A>G		
	ANO7	A>G		
	PMAIP1	A>G		
	PAQR5	C>T		
	ZYG11B	T>A		
	TSNAX	T>C		
	PHC3	G>T		
	TXNDC15	T>C		
	LYRM4	T>C		
	HOOK3	A>G		
	SMU1	G>T		
	SFXN2	T>C		
	SFXN2	A>T		2/7
	SFXN2	T>C		
	MPZL2	T>C		
	SCYL2	T>A		
	C14orf119	A>G		
	AXL	A>G		
	TANGO2	G>A		
	NFAM1	C>T		
	ZBTB8A	C>G		

Sample ID	Gene	Base change	rsID	Frequency (out of 7 total samples)
	TSNAX	G>C		
	TSNAX	G>A		
	TSNAX	G>A		
	IL17RD	C>T	rs141938030	
	IL17RD	T>C		
	ORAI2	A>C		
	LEP	A>G		
	LEP	C>T		
	KRTAP5-10	C>G		
	KRTAP5-10	A>T		
	IL18	G>T	rs191859038	
	STX2	C>T		
	MAPK1IP1L	A>G		
	LITAF	T>C		
	TMC7	T>C		
	ORC6	G>A		
	ORC6	C>T		
	CRLF3	A>G	rs9912283	
	PRR11	A>G		
	USP14	C>T		
	MAST3	G>A		
	NFAM1	G>T		
	NFAM1	C>G	rs41277493	

**Sample ID**	**Gene**	**Base change**	**rsID**	**Frequency (out of 7 total samples)**
	ZBTB8A	C>T		
	RSAD2	G>A		
	CEP68	A>G		
	CEP68	G>A		
	CEP68	T>C		
	TMEM169	A>G		
	LIMD1	A>G		
	DCP1A	T>C		
	DCP1A	T>C		
	NXPE3	T>C		
	LRRC58	A>G		
	PAK2	A>G	rs75759102	
	PAICS	A>G		
	LYRM7	A>G		
	CAMLG	A>T		
	FAM65B	G>A		
	FAM65B	T>G		
	ARMC10	T>A		
	EFCAB1	G>A		
	NDUFC2	C>T		
	TSPAN31	T>C	rs190042114	
	STX2	A>C		
	DNAL1	T>G		

Sample ID	Gene	Base change	rsID	Frequency (out of 7 total samples)
	CHP1	T>C		
	CHP1	G>A		
	CHP1	A>G		
	PDILT	T>C		
	FADS6	C>T		
	DSG2	A>C		
	QPCTL	G>A		
	QPCTL	G>C		
	QPCTL	T>C		
	ZNF587	T>A		
	CXorf36	G>C		
	CXorf36	T>C		
	WDR82	T>C		
	EMB	T>C		
	EMB	T>C	rs4095444	
	EMB	C>T	rs4095443	
	SKP1	G>A		
	SKP1	A>T		
	SFXN2	T>C		
	GOLGA3	C>T		
	DNAL1	G>A		
	DNAL1	T>C		
	TLK2	A>G	rs112287586	

Sample ID	Gene	Base change	rsID	Frequency (out of 7 total samples)
	PMAIP1	A>T		
	MAVS	A>G	rs11557298	
	ZNF785	T>C		
	RASSF2	A>G		
**3' Flank**	*BCL2L15*	T>C		4/7
	AQP6	C>T		
	FAM182B	A>G		
	GSTT1	T>A		3/7
	TMEM67	T>C		
	KIAA1324	C>T		2/7
	LINC00265	A>G		
	FAM182B	A>C		
	GSTT1	G>A		
	BCL2L15	G>T		
	TATDN3	A>G		
	GUCA1A	T>C	rs140024841	
	TMEM67	C>A	rs7009188	
	TCP11L1	G>A		
	KRT16P2	T>A		
	ZNF491	T>C		
	RP4-662A9.2	G>A		
	MRO	G>A		
	GSTT1	T>C		

Sample ID	Gene	Base change	rsID	Frequency (out of 7 total samples)
**5' UTR**	*C8orf46*	C>T	rs2555584	5/7
	ZNF683	T>C		4/7
	ING4	G>A		3/7
	C8orf46	A>G	rs12545784	2/7
	RASL11A	A>G		
	ZNF610	G>A		
	ZNF610	C>T		
	ZNF610	C>G		
**5' Flank**	*FAM225B*	C>T		6/7
	AP006285.6	G>T	rs582485	2/7
	SAA1	G>A	rs2445166	
	FAM225B	A>G	rs62574457	
**Intron**	*CD3G*	A>G		4/7
	DUOX1	G>A		
	BICC1	A>G		3/7
	ACSBG2	T>C	rs374402561	
	C11orf63	C>T		
	ZMYM1	C>T		
	NUTM1	C>A		2/7
	RP13-33H18.1	A>C		
	IGF2R	A>T		
	PSME2	T>A		
	LRP1B	A>G		

**Sample ID**	**Gene**	**Base change**	**rsID**	**Frequency (out of 7 total samples)**
	RP11-813N20.1	T>C		
	C11orf63	T>G		
	LPIN2	A>G		
	TPM4	T>A		
**Splice site**	*FCGR2A*	G>A	rs409763	2/7
	LINC00271	A>T		
