# Supplementary material for: Mutational landscape of head and neck squamous cell carcinomas in a South Asian population
Source: Genet Mol Biol. 2019 Nov 14;42(3):526–42. doi: 10.1590/1678-4685-GMB-2018-0005 (PMC6905448; doi:10.1590/1678-4685-GMB-2018-0005)
Supplement: Supplementary file 4 [file 1415-4757-GMB-42-3-2018-0005-suppl4.pdf]

Supplementary Material to "Mutational landscape of head and neck squamous cell carcinomas in a South Asian population"

Table S3 - Genes with somatic mutations in HNSCC patients resulting in nonsense or splice site variants.

Sample ID	Mutation type	Gene	Chr	Position	Base change	Amino acid change	Allele Frequency	COSMIC ID
NM-02	Nonsense	*SETD5*	chr3	9484995	C>T	R361X	0,167	COSM132643
		BTN3A3	chr6	26445970	G>T	E158X	0,231	-
		KLHL38	chr8	124659218	C>A	E463X	0,273	-
		DLG5	chr10	79614080	G>T	C195X	0,6	-
		FAM160A2	chr11	6239109	G>T	C583X	1	-
		DGKZ	chr11	46400648	C>G	Y1030X	0,273	-
		EED	chr11	85966291	G>T	G130X	0,111	-
		BIVM-ERCC5	chr13	103524719	G>A	W1404X	0,15	-
		SEL1L	chr14	81993290	C>A	E43X	0,231	-
		ADAM11	chr17	42850749	C>T	R316X	0,3	-
		CLASRP	chr19	45559754	C>A	Y142X	0,188	-
		TOP3B	chr22	22324643	C>A	E174X	0,15	-
	Splice site	*EIF4B*	chr12	53410256	G>T	-	0,2	-
		PSMB6	chr17	4700976	G>T	-	0,136	-
		MYCBPAP	chr17	48603241	G>T	-	1	-
NM-08	Nonsense	*ZAK*	chr2	174097092	G>T	E370X	0,214	-
		NCKIPSD	chr3	48717305	C>A	E427X	0,25	-
		FAT1	chr4	187540117	G>C	Y2541X	0,333	-
		FAM189A2	chr9	71990664	G>T	E46X	0,1	-

Sample ID	Mutation type	Gene	Chr	Position	Base change	Amino acid change	Allele Frequency	COSMIC ID
	Splice site	*FCGR2A*	chr1	161483723	G>A	-	0,111	-
**NM-11**	Nonsense	*CDC42BPA*	chr1	227216409	G>A	R1426X	0,143	-
		OXNAD1	chr3	16327942	A>T	K93X	0,154	-
		ABCC6	chr16	16280987	C>A	G621X	0,214	-
		RHPN2	chr19	33490585	G>A	Q378X	0,267	-
		RAD21L1	chr20	1210572	C>T	R65X	0,125	-
		USP9X	chrX	41029357	C>T	R916X	0,333	-
	Splice site	*RQCD1*	chr2	219457431	G>A	-	0,318	-
		BBS2	chr16	56544769	A>T	-	0,119	-
**NM-13**	Nonsense	*DDR2*	chr1	162725557	C>A	Y223X	0,214	-
	Splice site	*LPO*	chr17	56332334	T>G	-	0,182	-
		UPF1	chr19	18971634	G>T	-	0,667	-
**M-11**	Nonsense	*STAMBP*	chr2	74074655	G>T	E173X	0,206	-
		CDC27	chr17	45249321	G>T	C71X	0,214	-
		SYCP2	chr20	58486830	G>T	S316X	0,571	-
		KLHL13	chrX	117053561	G>A	Q168X	0,148	-
	Splice site	*CPSF3*	chr2	9583643	G>A	-	0,5	-
**M-12**	Nonsense	*COL19A1*	chr6	70610175	G>T	E71X	0,667	-
		KIAA0368	chr9	114156002	C>A	E1157X	0,176	-
		REC8	chr14	24648114	G>T	E398X	0,176	-
	Splice site	*OAZ3*	chr1	151736043	T>A	-	1	-
		CSTF3	chr11	33129547	C>A	-	0,231	-
		NUP107	chr12	69128614	G>A	-	0,143	-

**Sample ID**	**Mutation type**	**Gene**	**Chr**	**Position**	**Base change**	**Amino acid change**	**Allele Frequency**	**COSMIC ID**
		EIF4A3	chr17	78113806	C>A	-	1	-
**M-14**	Nonsense	*TMEM214*	chr2	27258844	C>G	S215X	0,1	-
		ETV7	chr6	36341284	C>A	G127X	1	-
		MYC	chr8	128750818	G>T	E119X	0,429	-
		GLI1	chr12	57858625	C>A	Y121X	0,097	-
		PTPN11	chr12	112892433	T>G	Y197X	0,143	-
		APOBR	chr16	28507408	C>A	S349X	0,429	-
		SS18L1	chr20	60733760	C>A	C34X	0,273	-
	Splice site	*FCGR2A*	chr1	161483723	G>A	-	0,133	-
		NAPB	chr20	23360081	C>G	-	0,098	-
