# Supplementary material for: Mutational landscape of head and neck squamous cell carcinomas in a South Asian population
Source: Genet Mol Biol. 2019 Nov 14;42(3):526–42. doi: 10.1590/1678-4685-GMB-2018-0005 (PMC6905448; doi:10.1590/1678-4685-GMB-2018-0005)
Supplement: Supplementary file 5 [file 1415-4757-GMB-42-3-2018-0005-suppl5.pdf]

Supplementary Material to "Mutational landscape of head and neck squamous cell carcinomas in a South Asian population"

Table S4 - Significant single nucleotide variants (SNVs) in HNSCC patients in non-coding regions**.**

SNV mutations identified by CADD score of ≥15

Region	Genes
3' UTR	*DUSP6*
	IGF1R
	ERBB4
	TES
	DPYSL2
Intron	*C16orf62*
	BAIAP2
	C5orf63
Splice site	*EIF2B4*
	PTPRA
